# A case report of laparoscopic orchopsis for transverse testicular ectopia via the Hesselbach route

**DOI:** 10.3389/fped.2025.1514662

**Published:** 2025-05-30

**Authors:** Xu Zhuofan, Zhao Xudong, Yuan Boxiang

**Affiliations:** Department of Pediatric Surgery, Zhongshan City People’s Hospital, Zhongshan, Guangdong, China

**Keywords:** transverse testicular ectopia, laparoscopic orchopsis, orchiopexy, Hesselbach route, Hei’s triangle

## Abstract

**Background:**

Testicular transverse ectopia (TTE) is a rare congenital abnormality in which both testicles descend into the same hemiscrotum via a solitary inguinal canal. This condition is frequently linked with inguinal hernias and various urogenital irregularities, and it may manifest as an unoccupied opposite scrotum or as a discernible lump in the groin or scrotal area.

**Patient presentation:**

A 9-month-old boy was diagnosed with a right inguinal hernia, which presented as a 3 cm × 3 cm palpable mass without pain upon examination. During surgery, both testicles were discovered to be undescended, fused by their ligaments, and had independent vas deferens, vascular supplies, and epididymides. The poorly developed testicles were fixed in the scrotum via Hei's triangle. Postoperative follow-up revealed normal testicular positioning, with no retraction, atrophy, or hernia recurrence.

**Conclusions:**

Orchiopexy via Hei's triangle is a new surgical option for patients with Type I TTE who have insufficient length spermatic cord blood vessels.

## 1 Introduction

Transverse testicular ectopia (TTE) is a rare anomaly characterized by the presence of both testes in the same hemiscrotum or both testes migrating and descending through a single inguinal canal (1). It can be associated with other anomalies, such as persistent Müllerian duct syndrome (PMDS), true hermaphroditism, inguinal hernia, hypospadias, and other urogenital abnormalities (2, 3). Following the first description by Von Lenhossek in 1886, it has been reported in >100 cases (4).

Transverse testicular ectopia not only separates from the optimal low-temperature environment for growth and development but also elevates the risk of infertility and malignancy (5, 6). The principal treatment of TTE is surgery. Some studies have shown that early intervention is most beneficial because it decreases the risk of testicular cancer and protects the spermatogenic capacity of the testis (7).

In this case study, we reported a case of laparoscopy orchiopexy for Type I transverse testicular ectopia via the Hesselbach route where the length of spermatic cord vessels is not enough.

## 2 Case report

A 9-month-old boy was referred to our pediatric surgery consultation for a right-sided inguinal hernia. According to his medical history, he was born at term and was hospitalized for neonatal intrauterine infection, myocardial damage, scalp hematoma, patent foramen ovale, and thalassemia during the postnatal period.

Inguinal ultrasound revealed a mixed echo mass in the right inguinal region, with a superior–inferior diameter of 25 mm and an anterior–posterior diameter of 19 mm. Peristalsis was visible within the mass, and no obvious fluid-filled dark area was observed. Color Doppler flow imaging revealed no significant abnormal blood flow signals were detected.

The bilateral testicle size and morphology were normal, with intact capsules, smooth surfaces, uniform internal echoes, and a normal blood supply. No abnormal echoes were observed in the bilateral epididymis. Fluid-filled dark areas were visible in the bilateral scrotal sacs, with a maximum thickness of 8 mm.

Physical examination revealed a mobile soft, reducible painless mass (3 cm × 3 cm) in the right inguinal region. After the mass entered the abdominal cavity and pressed the profoundly inguinal ring, the mass ceased to protrude during crying. Both sides of the testes were impalpable, and the scrotum was empty. No other anomalies were discovered.

**Figure 1:**
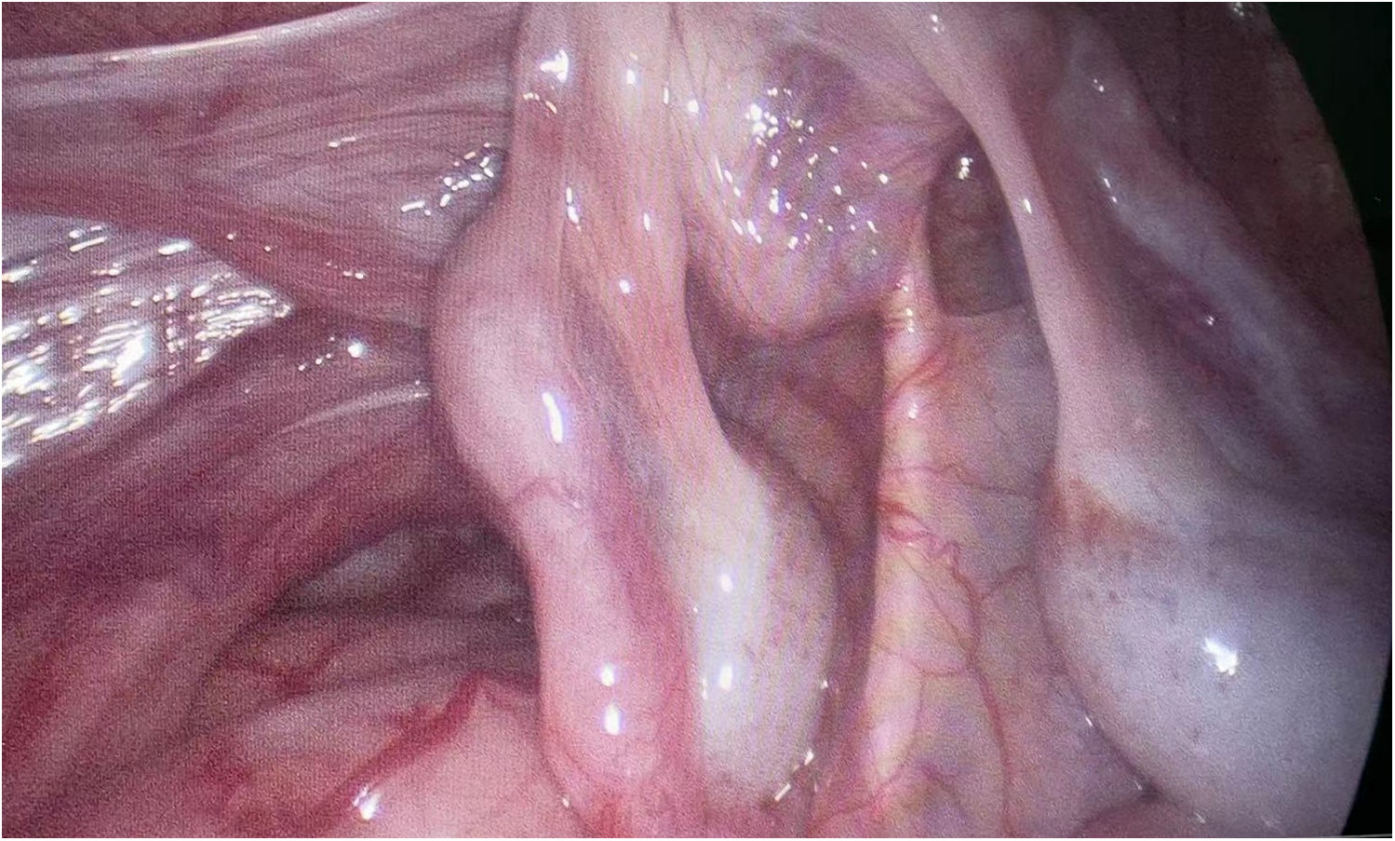
Both testes are poorly developed and have small volume.

During laparoscopy exploration, we incidentally discovered that both testes were located in the right inguinal abdominal ring. The testes were connected by a common gubernaculum, and each testis had an independent vas deferens supplying the vessel and epididymis. Both testes were poorly developed and had a small volume (0.8 cm  × 0.6 cm) (Figure 1).

**Figure 2:**
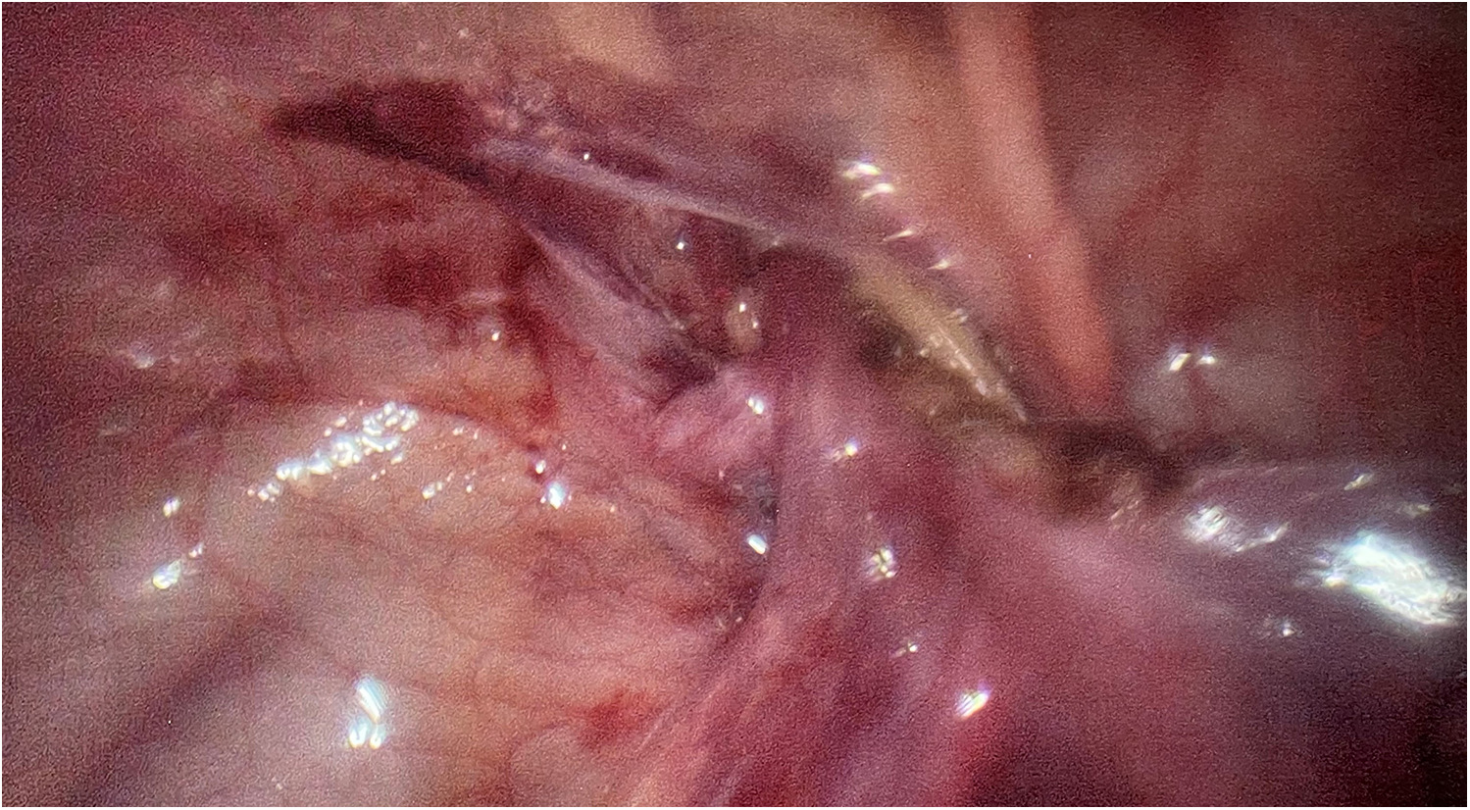
The left testis was brought down through the left Hesselbach canal.

**Figure 3:**
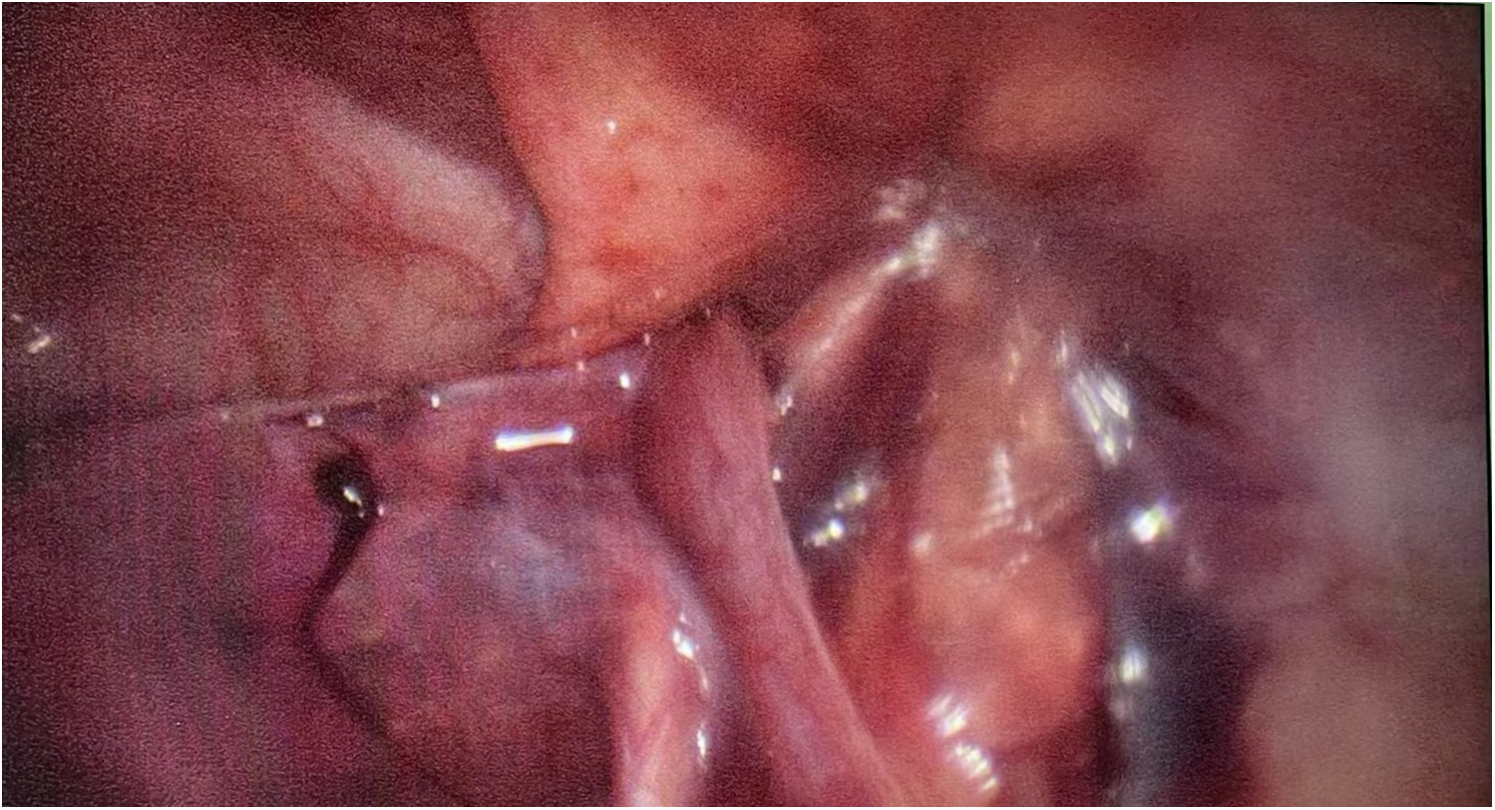
The right testis was brought down through the right Hesselbach canal.

The right spermatic cord entered the right internal inguinal ring, and the left testis had migrated to the right, pulling the spermatic cord externally (crossing testicular ectopia). Using laparoscopy, the crossing vas and vessels were dissected carefully after the covering peritoneum was incised. Both testes were brought down through the right/left Hesselbach canal and placed on their respective side of the scrotum (Figures 2, 3).

**Figure 4:**
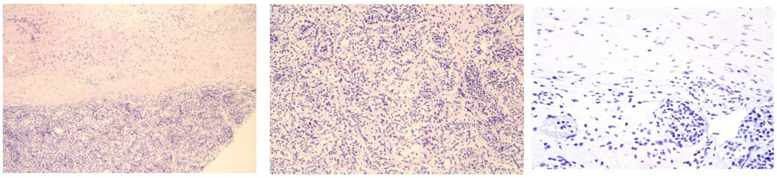
Pathological showed testicular tissue.

The pathological of the testes was testicular tissue (Figure 4), and the patient was discharged 4 days after the operation.

Three years of follow-up revealed no postoperative complications including testicular retraction and testicular atrophy. Follow-up color Doppler ultrasound showed the left testis measuring approximately 14 mm × 7 mm × 9 mm and the right testis approximately 15 mm × 7 mm × 9 mm. The left testis had a smooth surface with heterogeneous internal echotexture, showing scattered punctate hyperechoic foci, while the right testis demonstrated no abnormal echoes. Blood flow signals in both testes and epididymides were normal.

## 3 Discussion

TTE is also known as unilateral double testes, which is likely related to the inheritance of the disease in families (8), but the exact pathogenesis is not well understood (3). Only a few patients can be diagnosed before surgery, and most children are diagnosed during surgery. Based on the presence of various associated anomalies, TTE has been classified into three types (9):

• Type 1: accompanied only by hernia (40%–50%)
• Type 2: accompanied by persistent or rudimentary Müllerian duct structures (30%)
• Type 3: associated with disorders other than persistent Müllerian remnants (hypospadias, pseudohermaphroditism, and scrotal abnormalities) (20%).

Transverse testicular ectopia (TTE) is a rare form of testicular ectopia of uncertain embryological etiology. The adherence and fusion of developing Wolffian ducts, an aberrant gubernaculum, testicular adhesions, and traction on a testis by persistent Müllerian structures are examples of suggested embryological explanations. During fetal development, the mechanical effect of persistent Müllerian duct structures may prevent testicular descent and lead to both testicles descending toward the same hemiscrotum (10).

Our patient had Type I TTE. This patient presented with a reversible mass in the right inguinal region for 9 months. He had to be hospitalized in our hospital and undergo repeated physical examinations at our hospital, but he never experienced cryptorchidism because he had never sought medical advice from the pediatric surgery department. After the mass entered the abdominal cavity, both sides of the testes were impalpable, and the scrotum was empty. Preoperative color Doppler ultrasound revealed only a right indirect inguinal hernia, and both testicles were normal. Therefore, a right indirect inguinal hernia may contain a left testicle; when a hernia appears, the transverse testis meets the right scrotum. When hernia is reduced, the testes are also reduced into the abdominal cavity. Lacking experience, with interference from intestinal gas, it was difficult for ultrasound to display the correct testis position correctly.

The diagnosis of testicular torsion (TTE) relies primarily on physical examination and treatment, and US is a routine investigation performed prior to surgery. According to Kullendorff et al.'s study (11), ultrasound has an estimated sensitivity of 93% for detecting palpable testes; however, its sensitivity and specificity decrease to 45% and 78%, respectively, to accurately localize nonpalpable testes (12).

In this case, the undescended testis was accompanied by an inguinal indirect hernia, with the interference of intestinal gas, making it difficult for ultrasound to display clearly. When the undescended testis was retracted into the abdominal cavity and the hernia was reduced, the testis was pushed to a deeper position, which obviously interfered with the ultrasound results via the intestinal gas. Moreover, we did not examine and palpate the testis before the hernia retracted into the abdominal cavity, therefore leading to misdiagnosis. Finally, the correct diagnosis was made during laparoscopy exploration.

The most common options used during surgery are ectopic testis and blood vessels, vas deferens, and Müllerian duct remnants (including the uterus, valerian tubes, and upper two-thirds of the vagina). Surgical TTE has many options, including inguinal exploration and orchiopexy, diagnostic laparoscopy and orchiopexy, diagnostic laparoscopy and transseptal orchiopexy, diagnostic laparoscopy, and transseptal contralateral orchiopexy with congenital anomalies of repair at the end. Some authors have suggested fixing both testicles into the same hemiscrotum in cases where transseptal orchiopexy is not feasible (13).

The Hesselbach triangle consists of the lateral margin of the rectus abdominal muscle, the inferior abdominal artery, and the inguinal ligament. The lateral side is near the outer ring and the inguinal canal, and the inner side is near the medial umbilical ligament and bladder. In this case, the testicles were fixed on each side of the scrotum via the Hesselbach route. Compared with traditional routes, the Hesselbach route has more advantages, such as a shorter distance and wider path, which can effectively prevent blood vessel seizing, shorten the distance of testicular descent, and reduce the tension of spermatic cord traction.

Although the testes were successfully fixed through the Hesselbach route, some points still had to be attentive. First, this patient was so young that we did not perform TTE before surgery. During surgery, the testicles' blood vessels intersected with spermatic vessels, which made it difficult to separate the spermatic cord blood vessels. The length of the spermatic was relatively short; thus, it cannot be fixed by traditional orchiopexy. Second, because Hesselbach is close to the bladder, it is necessary to evacuate the bladder to separate the spermatic cord blood vessels. Third, we chose to use endoscopic separation forceps to establish a tunnel from the abdomen to the scrotum through the Hesselbach triangle, so we need more attention to avoid the occurrence of direct hernias caused by excessive tunnel width. After testis fixation, confirming the tension of the spermatic and spermatic blood vessels repeatedly to avoid excessive traction is necessary.

## 4 Conclusion

TTE is a rare condition that should be considered in cases with undescended testes. Patients can present with inguinal hernia on one side and cryptorchidism on the other side or with impalpable testis without any associated hernia, and the anomaly may be discovered incidentally during diagnostic laparoscopy for cryptorchism. Orchiopexy is necessary for Type I TTE patients. When the length of the spermatic cord vessels is insufficient, the testis can be pulled down through the Hesselbach canal. After surgery, the ectopic testicle has been repositioned to a relatively normal location, but there are still risks of scrotal trauma, testicular torsion, infertility, orchitis or epididymitis, and malignancy in the future, with the risk of malignant tumors at the rate of 18% (6). Therefore, long-term follow-up is necessary, and we recommend multiple follow-up examinations at postoperative 3 months, 6 months, 1 year, 2 years, 5 years, and puberty.

## Data Availability

The raw data supporting the conclusions of this article will be made available by the authors, without undue reservation.
